# Effect of sugarcane fiber digestibility, conservation method and concentrate level on the ruminal ecosystem of beef cattle

**DOI:** 10.1186/s13568-017-0356-7

**Published:** 2017-03-06

**Authors:** Johnny Maciel de Souza, Dannylo Oliveira de Sousa, Bruno Souza de Mesquita, Lígia Garcia Mesquita, Luis Felipe Prada Silva

**Affiliations:** 1 Department of Animal Science, School of Veterinary Medicine, Universidade de São Paulo, Pirassununga, São Paulo 13635-900 Brazil; 2 Ouro Fino Saúde Animal, Cravinhos, São Paulo 14140-000 Brazil

**Keywords:** Beef cattle, Ruminal fermentation, Ruminal microorganisms, qPCR, Sugarcane

## Abstract

The aim of this study was to investigate the effects of sugarcane neutral detergent fiber digestibility (NDFD), conservation method, and concentrate level on the ruminal microbial population of steers. Eight ruminal-cannulated Nellore steers were distributed in two contemporary 4 × 4 Latin Square design with a 2 × 2 factorial arrangement of treatments. Experiment 1: diets were formulated with 60% of concentrate level, and two sugarcane genotypes (high or low NDFD) either freshly cut or as silage. Experiment 2: diets were formulated with two levels of concentrate (60 or 80%), and two sugarcane genotypes (high or low NDFD) offered as freshly cut. Each experimental period lasted for 14 d, with the last 4 d used for ruminal fluid collection. Three cellulolytic bacteria (*Fibrobacter succinogenes*, *Ruminococcus albus*, *Ruminococcus flavefaciens*), two amylolytic (*Streptococcus bovis*, *Ruminobacter amylophilus*), and a lactate fermenting microorganism (*Megasphaera elsdenii*) were quantified by qPCR. Experiment 1: diets with fresh sugarcane increased the population of *S. bovis*, and *M. elsdenii*. Sugarcane with high NDFD increased *F. succinogenes* population only when sugarcane was offered as freshly cut. Experiment 2: increasing concentrate in the diet decreased *S. bovis* population, and increased *R. amylophilus*. Sugarcane with high NDFD increased the population of cellulolytic bacteria only at the 60% concentrate diet. Providing sugarcane with high NDFD favored the growth of fibrolytic bacteria, and this effect were dependent on the conservation method and on diet concentrate level. In addition, sucrose appears to have great effect on the composition of ruminal microflora, especially *S. bovis*.

**Electronic supplementary material:**

The online version of this article (doi:10.1186/s13568-017-0356-7) contains supplementary material, which is available to authorized users.

## Introduction

Feedlot diets have high inclusion of concentrate and low roughage, usually resulting in low ruminal pH that directly affects the digestibility of the fiber, and may also lead to metabolic disorders (Anderson et al. 2016). *Fibrobacter succinogenes*, *Ruminococcus flavefaciens* and *Ruminococcus albus* are considered the main bacteria responsible for roughage fiber degradation (Forsberg et al. 1997), and the proliferation of these microorganisms in the rumen is directly correlated with the quality and quantity of dietary fiber. Replacing fiber with non-fibrous carbohydrates (NFC), such as starch and sugars, influences the development of cellulolytic microorganisms, changing the dynamics of fermentation in the ruminal ecosystem (Tajima et al. 2001).

As the concentration of fermentable carbohydrates in the rumen increases, microbial growth is stimulated, increasing ruminal fermentation rate. On the other hand, increasing the digestibility of carbohydrates in the rumen results in pH to drop below 6.0 due to the greater production of short-chain fatty acids (SCFA). In this situation, there is a reduction in the activity of fibrolytic bacteria, affecting the digestibility of fiber, while there is increased activity of amylolytic bacteria, such as *Streptococcus bovis* and *Ruminococcus amylophilus*, and lactate users, as *Megasphaera elsdenii* (Tajima et al. 2001; Nagaraja and Titgemeyer 2007; Petri et al. 2012). The excess of ruminal fermentation of NFC, combined with low inclusion of roughage in the diet, can lead to acidosis, decreasing fiber digestibility, dry matter intake and, consequently, causing a reduction in weight gain of animals (Gonzalez et al. 2012).

Therefore, one alternative would be to use better quality roughages, reducing the diet concentrate content and favoring the proper development of cellulolytic microorganisms. Inclusion of high quality forage in the diet is positively related to growth of cellulolytic bacteria populations in the rumen (Fernando et al. 2010; Koike and Kobayashi 2001). Among the roughages, sugarcane is widely used as forage in diets for ruminants, either as freshly cut or ensiled (Freitas et al. 2006). However, beside low protein content, poor fiber digestibility is the main limitation of animal performance in sugarcane-based diets (Pereira et al. 2001). Moreover, sugarcane has high sucrose content, and despite the importance of fiber digestibility for animal performance, the sugarcane breeding programs are focused on total production of sucrose, with little attempt to evaluate quality parameters for animal nutrition. Forage breeding demonstrates that small increases in dry matter (DM) digestibility result in significant improvement in animal performance (Casler 2001).

In this context, it is important to understand how neutral detergent fiber digestibility (NDFD) of sugarcane genotypes, the level of concentrate, and the method of conservation affects the population dynamics of ruminal microorganisms. The ensiling process changes the chemical composition of sugarcane, increasing fiber and reducing the sucrose content (Kung and Stanley 1982; Daniel et al. 2013), altering the ruminal fermentation (Hungate 1966; Vallimont et al. 2004; Singh et al. 2014). These factors directly affect the mechanisms involved in fiber degradation by ruminal microorganism. Therefore, the objectives of this study were to evaluate the effect of NDFD and conservation method of sugarcane, and the concentrate level in the diet on the population of ruminal bacteria of cannulated beef cattle.

## Materials and methods

All experimental procedures were in agreement with the Guide for Care and Use of Agricultural Animals in Agricultural Research and Teaching (FASS 1999), with all animal procedures approved by the University of São Paulo Animal Bioethics Committee (Protocol number 7632051113).

For this experiment, samples of rumen contents coming from two previous experiments were used (Mesquita 2013; Sousa et al. 2014), which presented the results of performance and ruminal kinetics. The experiments were conducted at the College of Veterinary Medicine and Animal Science, University of São Paulo Beef Cattle Research Laboratory (Pirassununga, SP, Brazil). In both developed experiments, rumen contents samples were collected from cannulated animals for quantification on the ruminal microorganisms of interest.

### Animals and diets

In the first experiment (Sousa et al. 2014), eight ruminal-cannulated Nellore steers were distributed in two contemporary 4 × 4 Latin Square design. Experimental diets were formulated with 40% sugarcane, either freshly cut or as silage, and 60% concentrate on a DM basis. As roughage, two genotypes with high or low digestibility of NDF (NDFD) were used, making up 4 experimental diets in a 2 × 2 factorial arrangement of treatments (2 modes of conservation and 2 NDFD).

In the second experiment (Mesquita 2013), eight ruminal-cannulated Nellore steers were distributed in two contemporary 4 × 4 Latin square design. Experimental diets were formulated with 40 or 60% of fresh sugarcane, and two levels of concentrate on a DM basis. As roughage, two genotypes with high or low NDFD were used, making up four experimental diets in a 2 × 2 factorial arrangement of treatments (two levels of concentrate and two NDFD).

In both experiments, the sugarcane genotypes used were IAC86-2480 (high NDFD) and SP91-1049 (low NDFD). The genotype IAC86-2480 was confirmed as having 20.5% greater NDFD than the genotype SP91-1049 (38.5 vs. 32.0 ± 1.1%). The diets were formulated according to NRC (1996), aiming a weight gain of 1.2 kg/day. The concentrate was composed of ground corn (46.7% DM basis), soybean meal (10% DM basis), urea (1.24% DM basis), limestone (0.50% DM basis), salt (0.25% DM basis) and a commercial mineral supplement (1.3% DM, Minerthal MD ^®^). The diets were fed ad libitum twice daily, and the control of feed was done individually, by daily weighing of food provided and remains, allowing between 5 and 10% of orts. The steers were kept in tie stalls with concrete floors, individual feeders, and water bunks. No bedding was used. Stalls and bunks were cleaned daily. A detailed description of the diets can be found at Mesquita (2013) and Sousa et al. (2014).

Each experimental period lasted 14 days, with 10 days for diet adaptation and the last 4 days used for determination of consumption (dry matter intake, DMI; and indigestible neutral detergent fiber, iNDF), ruminal evacuation and collection of ruminal fluid. Steers were weighed weekly for 2 consecutive days, without water or feed restriction.

### Collection and processing of rumen contents

For both experiments, the ruminal content was evacuated manually through the rumen cannula in fasted animals (0 h after feeding) and 4 h after eating. The total mass of ruminal content and volume were determined. During the evacuation, an aliquot of 10% was separated for sub-sampling facility. 600 mL of a representative sample of the liquid and solid phase (proportional) was used to determination of the bacterial population, and the unused rumen contents were returned to each steer via the fistula. These samples were stored properly in a freezer at −20 °C.

For each sample, 25 mL of fluid and 25 g of solids were used for processing, as described by Stevenson and Weimer (2007), and the resulting bacteria pellet was dissolved in 700 μL of buffer (100 mM Tris/HCl, 10 mM EDTA, and 0.15 M NaCl, pH 8.0) and stored at −80 °C until DNA extraction.

### qPCR of ruminal bacteria

Three cellulolytic bacteria (*F. succinogenes*, *R. albus* and *R. flavefaciens*), two amylolytic (*S. bovis* and *Ruminobacter amylophilus*), and a lactate fermenting microorganism (*M. elsdenii*) were quantified by the technique of qPCR to determine the effect of diet on the population of ruminal microorganisms.

DNA extraction was performed for each sample of rumen content, with QIAamp DNA Stool Mini Kit (Qiagen, Valencia, CA) and used according to the manufacturer’s instructions. For extraction, 200 μL of ruminal content per sample were used. Based on the study of Henderson et al. (2013), although the total yield of DNA from this particular kit was lower than extraction with phenol chloroform-based methods, it produces DNA with good quality for PCR amplification from cow rumen contents. The real-time qPCR reactions were performed in 96-well plates on a 7500 Real Time PCR System (Applied Biosystems^®^, Life Technologies, Foster City, CA) for each ruminal sample individually, containing 10 μL of 2× SYBR Green Master Mix (Applied Biosystems^®^, Life Technologies, Foster City, CA), 1.2 μL of primer Forward and 1.2 μL of primer Reverse for respective bacteria (Table 1), 6.6 µL of MilliQ water, and 1 μL of DNA template in a final volume of 20 μL per reaction.

**Table 1:** Real-time PCR primers used in the relative quantification of ruminal microorganisms

Species	Sequence (5′–3′)	Amplicon size (bp)	Reference
*F. succinogenes*	F: GGTATGGGATGAGCTTGC R: GCCTGCCCCTGAACTATC	445	Tajima et al. (2001)
*R. albus*	F: CCCTAAAAGCAGTCTTAGTTCG R: CCTCCTTGCGGTTAGAACA	175	Wang et al. (1997)
*R. flavefaciens*	F: TCTGGAAACGGATGGTA R: CCTTTAAGACAGGAGTTTACA	295	Koike and Kobaiashi (2001)
*S. bovis*	F: CTAATACCGCATAACAGCAT R: AGAAACTTCCTATCTCTAGG	127	Stevenson and Weimer (2007)
*R. amylophilus*	F: CAACCAGTCGCATTCAGA R: CACTACTCATGGCAACAT	642	Tajima et al. (2001)
*M. elsdenii*	F: GACCGAAACTGCGATGCTAGA R: CGCCTCAGCGTCAGTTGTC	129	Ouwerkerk et al. (2002)
*Eubacteria*	F: CCTACGGGAGGCAGCAG R: ATTACCGCGGCTGCTGG	193	Muyzer et al. (1993)

*F* forward, *R* reverse

A universal primer was used for quantification of total bacteria (Table 1) for each ruminal sample individually, to standardize the amount of DNA added to the reactions, following the same qPCR conditions describe above. The qPCR reactions of all bacteria were run in duplicate, and a negative control was included in each assay to assess the specificity of qPCR reaction.

The qPCR amplification protocol was as follows: an initial denaturation step at 95 °C for 10 min, then 44 cycles of heating and cooling at 95 °C for 15 s, followed by annealing step at 60 °C for 30 s, and extension at 72 °C for 30 s. Melting curves were analyzed at the end of the reactions to verify the specificity of each amplification.

### Statistical analysis

Data from the two experiments were analyzed separately. The data were analyzed in a contemporary Latin square design, using the MIXED procedure of SAS version 9.3, which previously verified the normality of residuals by the Shapiro–Wilk’s test and the homogeneity of variances compared by Levene’s test. The data of relative quantification of ruminal bacteria by qPCR were analyzed by analysis of covariance, according to the model of relative expression proposed by Yuan et al. (2006). For the first experiment, the model included the fixed effects of sugarcane neutral detergent fiber digestibility (NDFD), conservation method (CONS), and their interaction. The square effect, animal inside square and period were included as random factors in the model. For the second experiment, the model included the fixed effects of level of concentrate (DIET), sugarcane NDF digestibility (NDFD), and their interaction. The square effect, animal inside square and period were included as random factors in the model. In both models, the repeated measures factor was included, relating to different times of rumen content collection. The degrees of freedom and tests were adjusted using the Kenward-Roger option. Treatment means were compared with the least significant difference and significance declared at the 0.05 probability level. When significance of the interaction between the main effects was ≤0.10, the interaction was decomposed and the treatments effects were analyzed by using the SLICE option of PROC MIXED. Data is presented as percentage of total microbial population.

## Results

### Study 1

In the first study, the effect of fiber digestibility on *F. succinogenes* population was dependent on the forage conservation method, as there was a significant NDFD × CONS interaction (*P* = 0.01; Table 2). Feeding diets containing the high-NDFD sugarcane genotype increased (*P* < 0.01) *F. succinogenes* population, but only when fed as fresh sugarcane and not when fed as silage.

**Table 2:** Relative population of ruminal microorganisms in function of digestibility of neutral detergent fiber (high or low NDFD) and conservation method (fresh or silage) of sugarcane, considering the means of two collection times

Species	Fresh		Silage		SEM	*P* value		
	Low NDFD^1^	High NDFD^2^	Low NDFD^1^	High NDFD^2^		NDFD	Cons	NDFD*Cons
*F. succinogenes*	0.014^a^	0.036^a^	0.023^ab^	0.009^b^	0.050	0.98	0.22	0.01
*R. albus*	0.002	0.004	0.001	0.002	0.004	0.12	0.17	0.91
*R. flavefaciens*	0.013	0.008	0.006	0.005	0.006	0.37	0.27	0.59
*S. bovis*	0.025	0.015	0.003	0.003	0.009	0.53	<0.01	0.47
*R. amylophilus*	0.013	0.009	0.013	0.007	0.004	0.13	0.82	0.77
*M. elsdenii*	0.102	0.053	0.012	0.013	0.075	0.61	0.06	0.51

The relative population size is presented as percentage of total microbial population

*NDFD* effect of NDF digestibility, *Cons* effect of conservation method

^a,b^Means within a row with different superscript letters differ, *P* < 0.05

^1^SP91-1049 sugarcane genotype

^2^IAC86-2480 sugarcane genotype

The sugarcane genotype, as well as the conservation method, did not influence the population of *R. albus* (*P* = 0.12 and *P* = 0.17, respectively), and *R. flavefaciens* (*P* = 0.37 and *P* = 0.27, respectively). There was no significant interaction between the factors described for both ruminal microorganisms (*P* = 0.91 and *P* = 0.59, respectively).

The sugarcane genotype did not affect the population of *S. bovis* (*P* = 0.53) and *M. elsdenii* (*P* = 0.61). However, the supply of sugarcane as freshly cut increased the population of *S. bovis* (0.019 vs 0.003%, *P* < 0.01) and *M. elsdenii* (0.073 vs 0.013%, *P* = 0.06) when compared to feeding sugarcane as silage (Table 2).

Ruminal content collection time also affected the relative population of ruminal bacteria. There was a reduction in *F. succinogenes* relative population after feeding (*P* = 0.03), whereas the relative population of *R. amylophilus* (*P* < 0.01) and *M. elsdenii* (*P* < 0.01) was greater 4 h after feeding (Table 3).

**Table 3:** Relative population of ruminal microorganisms, in function of time of collection of ruminal content, of both studies

Species	Study 1		SEM	*P* value	Study 2		SEM	*P* value
	0 h	4 h			0 h	4 h		
*F. succinogenes*	0.024	0.013	0.005	0.03	0.010	0.012	0.004	0.57
*R. albus*	0.002	0.002	0.001	0.54	0.004	0.003	0.002	0.77
*R. flavefaciens*	0.007	0.009	0.002	0.31	0.012	0.016	0.004	0.31
*S. bovis*	0.008	0.007	0.002	0.55	0.014	0.006	0.003	<0.01
*R. amylophilus*	0.005	0.021	0.003	<0.01	0.008	0.032	0.005	<0.01
*M. elsdenii*	0.017	0.054	0.009	<0.01	0.098	0.251	0.046	<0.01

The relative population size is presented as percentage of total microbial population

### Study 2

In the second study, the effect of fiber digestibility on *F. succinogenes*, *R. albus* and *R. flavefaciens* populations was dependent on the concentrate level of the diet, as there was a significant NDFD × Diet interaction (*P* = 0.06, *P* < 0.01, and *P* = 0.09 respectively; Table 4). Feeding diets containing the high-NDFD sugarcane genotype increased the populations of *F. succinogenes* (*P* < 0.01) and of *R. albus* (*P* < 0.01), but only in the diet with 60% concentrate, and not in the 80% concentrate diet. However, the opposite effect was observed for the *R. flavefaciens* population, where feeding the high-NDFD sugarcane genotype increased (*P* < 0.01) *R. flavefaciens* population only at the 80% concentrate diet.

**Table 4:** Relative population of ruminal microorganisms in function of digestibility of neutral detergent fiber (high or low NDFD) of fresh sugarcane and level of concentrate in diet (60 or 80%), considering the means of two collection times

Species	60% concentrate		80% concentrate		SEM	*P* value		
	Low NDFD^1^	High NDFD^2^	Low NDFD^2^	High NDFD^2^		NDFD	Diet	NDFD*Diet
*F. succinogenes*	0.012^b^	0.023^a^	0.010^b^	0.006^b^	0.005	0.74	0.02	0.06
*R. albus*	0.002^b^	0.012^a^	0.004^ab^	0.002^b^	0.003	0.21	0.20	<0.01
*R. flavefaciens*	0.012^ab^	0.008^b^	0.013^ab^	0.031^a^	0.007	0.49	0.07	0.09
*S. bovis*	0.024	0.019	0.004	0.005	0.008	0.94	<0.01	0.57
*R. amylophilus*	0.015	0.009	0.021	0.021	0.006	0.47	0.07	0.37
*M. elsdenii*	0.145	0.070	0.237	0.254	0.106	0.38	0.25	0.29

The relative population size is presented as percentage of total microbial population

*NDFD* effect of NDF digestibility (NDFD), *Diet* effect of level of concentrate in diet

^a,b^Means within a row with different superscript letters differ, *P* < 0.05

^1^SP91-1049 sugarcane genotype

^2^IAC86-2480 sugarcane genotype

Furthermore, the increase of concentrate in the diet, from 60 to 80%, resulted in a significant reduction in the population of *S. bovis* (0.022 vs 0.004%, *P* < 0.01). On the other hand, there was an increase in population of *R. amylophilus* (0.012 vs 0.021%, *P* = 0.07) in response to the increase of concentrate in the diet. There were no effects of the diet, NDFD or the interaction between these factors on the population of *M. elsdenii* (*P* > 0.10).

Regarding the time of rumen content collection, there was a reduction in the population of *S. bovis* 4 h after feeding (*P* < 0.01), whereas the population of *R. amylophilus* (*P* < 0.01) and of *M. elsdenii* (*P* < 0.01) was greater 4 h after feeding (Table 3).

## Discussion

Forage quality is an important factor regulating intake and efficiency of diet utilization, as well as in reducing the amount of concentrate in the diet of ruminants (Tafaj et al. 2005). Sugarcane has a particular physiological maturation process, as with the advance of maturity there is a decrease in fiber digestibility and in CP content. However, because of sucrose accumulation, there is an increase in NFC content leading to increase the total DM digestibility as maturity progress (Kung and Stanley 1982; Carvalho et al. 2010). However, the effect of sugar, like sucrose, on ruminal microorganisms has not been extensively studied (Sun et al. 2015). Therefore, low fiber digestibility is usually the main limiting factor for high performance beef cattle fed with sugarcane based diets, as NDF digestibility in sugarcane can be about half of that of corn silage (Corrêa et al. 2003).

The proportion of high quality forage in the diet exerts a positive influence on growth of cellulolytic bacteria in the rumen (Fernando et al. 2010), increasing intake and, consequently, animal performance. Thus, we hypothesized that modulation of animal performance by diet could be explained by modulation of the population of ruminal microorganisms. In the present study, the use of a sugarcane genotype with high-NDF digestibility favored growth of fibrolytic bacteria in the rumen, likely by substrate availability and maintenance of optimum rumen pH. To our knowledge, no other study evaluated the change in ruminal bacteria population to differing fiber digestibility of the roughage.

It is important to consider that in the present study, both liquid and solid fractions were analyzed together, which could potently mask important population changes happening in each of the fractions separately. Also, the method of DNA extraction can also influence the results, although the variation between DNA extraction methods is usually smaller than the variation caused by changes in diet (Henderson et al. 2013).

In the present study, the two different sugarcane genotypes had different chemical composition. The lignin and indigestible FDN contents were lower for the high-NDFD genotype, with greater DM and NDF digestibility (Sousa et al. 2014; Mesquita 2013). In the first study, the effect of greater NDF digestibility of sugarcane on *F. succinogenes* population was dependent on the method of conservation. *F. succinogenes* was increased with high-NDF digestibility, when sugarcane was offered as freshly cut. The *R. albus* population followed a similar pattern, although not significant (*P* = 0.12). The silage process alters the nutritional quality of roughages, where part of the NFC is consumed, and causing increase in the NDF and lignin concentration (Kung and Stanley 1982). Mode of conservation changed the chemical composition of sugarcane, with greater lignin and iNDF in the ensiled sugarcane, while DM and NDF digestibility were greater for the freshly-cut sugarcane. Therefore, when sugarcane is offered as freshly cut there is more soluble sugar arriving in the rumen environment. Consequently, mode of conservation influenced rumen pH, with greater rumen pH when sugarcane was offered as silage than as freshly cut (6.69 vs. 6.37 ± 0.08; *P* < 0.01). There was no effect of sugarcane genotype on rumen pH levels (Sousa et al. 2014). According to Tafaj et al. (2005), a moderate quantity of NFC in the diet can stimulate fiber digestibility because of better supply of fermentable organic matter, nitrogen, and energy for the ruminal bacteria.

Among the cellulolytic bacteria present in the rumen, *F. succinogenes, R. flavefaciens,* and *R. albus* are considered the main species responsible for fiber degradation (Varel and Dehority 1989). Therefore, proliferation of cellulolytic bacteria in the rumen would be correlated with the amount of digestible fiber in the diet, and the substitution of fiber for soluble carbohydrates would influence its growth and alter the dynamic of the rumen ecosystem (Tajima et al. 2001). In the second experiment, the effect of high-NDF digestibility on the population of these three species of fibrolytic bacteria was dependent on the level of concentrate in the diet; where the population of *F. succinogenes* and *R. albus* responded to high-NDF digestibility only at the lower level of concentrate inclusion. However, *R. flavefaciens* population responded to high-NDFD only in the diet with 80% concentrate. In study 2, rumen pH was influenced only by concentrate level, where the 60% concentrate diet had greater mean rumen pH than the 80% concentrate diet (6.38 vs. 6.12 ± 0.11, *P* < 0.05, Mesquita 2013). Although the three studied species *F. succinogenes, R. flavefaciens,* and *R. albus* are considered important rumen cellulolytic bacteria, their mechanism of action, ability to adhere to particles and enzymatic profiles are different (Mosoni et al. 1997). When cultured with excess cellulose, the number of cells for these three species was similar (Shi et al. 1997). However, when cultured with limited amount of cellulose, *R. flavefaciens* predominated over the other two, demonstrating the superior adhesion capacity for this microorganism (Shi et al. 1997). In the context of study 2, diets with the high-NDFD sugarcane genotype increased the total intake, NDF rumen passage rate and body growth of the animals, but only in the diet with 80% concentrate (Mesquita 2013). Also, the diet with 80% concentrate reduced rumen ammonia levels (11.66 vs. 7.18 mg/dL for the 60 and 80% concentrate diets, respectively—Mesquita 2013).

With the increment of fermentable carbohydrates in the rumen, there is an overall stimulus for microbial fermentation. On the other hand, rapidly fermentable carbohydrates and the accumulation of SCFA in the rumen forces a decline in ruminal pH, usually for values bellow 6.0. In these situations, there is a reduction in the activity of fibrolytic bacteria, hindering fiber digestibility (Weimer 1996; Owens et al. 1998; Russell and Rychlik 2001) while simultaneously stimulating amylolitic and lactate utilizing-bacteria (Tajima et al. 2001; Nagaraja and Titgemeyer 2007). Besides direct inhibition of cellulolytic bacteria with low ruminal pH, there is also a decline in attachment of bacteria to the substrate, caused by the lack of positive effectors, such as the ion bicarbonate, and by the excess of attachment inhibitors, such as soluble starch (Owens and Goetsch 1993).

Petri et al. (2012) observed that great inclusion of concentrate in the diet, in substitution for the roughage, reduces the populations of the fibrolytic bacteria *F. succinogenes*, *R. flavefaciens*, and *R. albus*. The substitution of roughage for concentrate, rich in rapidly fermentable carbohydrates, promoted a decline in particle size of the diet, with less physically effective NDF, reducing salivation and rumen motility and, consequently, rumen buffering. Several other studies demonstrate the reduction in cellulolytic bacteria due to the increase in concentrate in the diet (Tajima et al. 2001; Singh et al. 2014; Granja-Salcedo et al. 2016).

Different from the fibrolytic bacteria, the amilolytic bacteria (represented in this study by *R. amylophilus* and *S. bovis*) have preference for NFC and are more tolerant to low pH. In the present study, there was an increase in the population of *R. amylophilus* with greater concentrate inclusion in the diet, reflecting the increase in starch and total NFC in the diet (Schwartzkopf-Genswein et al. 2003; Petri et al. 2012). However, the *S. bovis* population was reduced with greater concentrate in the diet, and was increased with freshly cut sugarcane compared to sugarcane conserved as silage. It is important to highlight that the roughage used in this study was sugarcane, with high level of soluble sugars (mainly sucrose). Also, sugarcane as silage has lower sugar content than freshly cut sugarcane. Therefore, in the present study, the treatments that provided more soluble sugar and less starch to the rumen (lower concentrate inclusion and freshly cut sugarcane) favored growth of *S. bovis*. Hence it can be suggested that *S. bovis* prefer NFC sources other than starch, such as sucrose.

Supporting this hypothesis, Golder et al. (2014) reported that *S. bovis* became more prevalent in heifers fed with fructose than with starch. Moreover, the relative abundance of the *Streptococcaceae* and *Veillonellaceae* families was increased when heifers received fructose. Other studies found similar results, where the population of *S. bovis* was not increased with the increment of concentrate in the diet of Nellore steers, using corn silage as the roughage source (Granja-Salcedo et al. 2016) or freshly-cut sugarcane (Ribeiro Junior et al. 2016). Sun et al. (2015) report an increase of *S. bovis* population by replacing cornstarch in a high-concentrate diet (forage to concentrate ratio = 40:60) with 3% sucrose after 24 h in vitro incubation.

The ruminal bacteria *S. bovis* is a facultative anaerobe and tolerant to low pH, and is known to be prevalent during lactic acidosis, proliferating in the rumen of cattle fed high levels of concentrate (Owens et al. 1998; Russell and Hino 1985; Russell and Rychlik 2001). However, the fast growth of *S. bovis* was not observed in animals adapted to starch rich diets, but in animals with lactic acidosis (Nagaraja and Titgemeyer 2007). Fernando et al. (2010) reported that the population of *S. bovis* increased at the beginning of the adaptation regimen to high grain diet, whereas at the end of the step-up adaptation, the *S. bovis* population decreased, and did not show a significant change in population size compared to control, where animals received only hay during the adaptation phase.

Therefore, whether the animals are adapted or not to a high-grain diet seems to influence the effect of addition of grains in the *S. bovis* population in the rumen. The abrupt change in the diet in non-adapted animals favors the rapid growth of *S. bovis*, lactate and SCFA accumulation, decline in rumen pH, and metabolic disorders (Russell and Hino 1985; Hernandez et al. 2014). In contrast, when animals are adapted to high-grain diets, the *S. bovis* population can be controlled favoring the growth of lactate utilizers, such as *M. elsdenii* and *Selenomonas ruminantium*, and the maintenance of rumen pH (Fernando et al. 2010). Hence, the importance of removal of fermentation acids, specially lactate, so as not to cause the decline in rumen pH.

Some microorganism present in the rumen and tolerant to low pH can utilize lactate, therefore avoiding lactate accumulation in the rumen environment. The lactate utilizing bacteria measured in the present study was *M. elsdenii*, which population increased when sugarcane was offered as freshly cut as opposed to sugarcane silage. This microorganism is able to grow on sucrose or glucose, although from these substrates the end product is butyrate, not propionate which comes from lactate (Hino et al. 1994). In the study 2, there was greater proportion of butyric acid in the rumen of animals receiving diets with 60% concentrate in comparison to 80% of concentrate (15.67 vs. 13.28% of total SCFA, *P* = 0.01—Mesquita 2013), and this can be explained by the action of *M. elsdenii*. This increase in molar proportion of butyrate in response to the partial substitution of starch with sugar was consistent to previous studies (Vallimont et al. 2004; Sun et al. 2015).

Khafipour et al. (2009) reported that *S. bovis* was the prevalent species during severe acidosis and that *M. elsdenii* was the dominant species during mild acidosis caused by excess of grain in the diet. Also, strains of *M. elsdenii* has been used to reduce lactate accumulation and increases rumen pH, consequently preventing ruminal acidosis (Long et al. 2014). In the present study, the diet with fresh sugarcane provided more sucrose to the rumen, favoring rumen fermentation and more acid accumulation, including lactate. The transient increase in lactate would favor growth of *M. elsdenii*.

It is clear that NFC supply to the rumen alter the fermentation profile and therefore the ruminal microorganism populations. This fact becomes evident when the different sampling times are considered. When rumen samples were collected shortly after feeding, when there is a great supply of NFC to the rumen, there was a reduction in the population of *F. succinogenes* and an increase in the populations of *R. amylophilus* and *M. elsdenii*. Similar results have been reported in ovine by Mosoni et al. (2007), with a reduction in the populations of fibrolytic bacteria (*F. succinogenes, R. albus,* and *R. flavefaciens*) when the rumen was sampled 3 h after feeding. The population of another lactate utilizing bacteria, *S. ruminantium*, was also increased 2 h after feeding (Singh et al. 2014). The rumen is a complex and dynamic environment where the microorganisms must constantly adapt to changes in diet composition, amount and frequency of feeding.

Therefore, the supply of sugarcane with greater NDFD favored the growth of fibrolytic bacteria, but this effect was dependent on the conservation method and the concentrate level. The genotype with higher NDFD favored the growth of *F. succinogenes* only when sugarcane was offered as freshly cut. Furthermore, on a diet with less addition of concentrate, the population of fibrolytic bacteria *F. succinogenes* and *R. albus* increased in response to increasing NDFD. When sugarcane was offered as freshly cut, the population of *S. bovis* and *M. elsdenii* were increased in response to greater supply of readily fermentable sugars. In addition, the increase of dietary concentrate increased the population of *R. amylophilus* and reduced *F. succinogenes*, *R. albus* and *S. bovis*, highlighting the hypothesis that *S. bovis* has a higher affinity for sugar (Additional file 1) than the for starch itself.

## Additional file

**Additional file 1.** Results of qPCR amplification of *F. succinogenes*, *R. flavefaciens*, *R. albus*, *S. bovis*, *R. amylophilus*, and *M. elsdenii*.

## Abbreviations

NDFD: neutral detergent fiber digestibility
NDF: neutral detergent fiber
qPCR: quantitative polymerase chain reaction
NFC: non-fibrous carbohydrates
SCFA: short-chain fatty acids
DM: dry matter
DMI: dry matter intake
iNDF: indigestible neutral detergent fiber
CONS: conservation method
DIET: level of concentrate
